# NECAB3 promotes the migration and invasion of liver cancer cells through HIF-1α/RIT1 signaling pathway

**DOI:** 10.1515/med-2023-0700

**Published:** 2023-05-16

**Authors:** Yicheng Tian, Longjiang Shao, Qi Wang, Gan Ru, Chuntao Zhai, Lihui Zhou

**Affiliations:** Department of General Surgery, Suzhou Science & Technology Town Hospital, Gusu School, Nanjing Medical University, No. 1 Lijiang Road, Huqiu District, Suzhou, Jiangsu, 215153, China; Department of General Surgery, Suzhou Science & Technology Town Hospital, Gusu School, Nanjing Medical University, Suzhou, Jiangsu, 215153, China

**Keywords:** NECAB3, HIF-1α, RIT1, liver cancer, migration, invasion

## Abstract

Liver cancer is a prevalent malignant tumor with high mortality worldwide, making it urgent to explore new targets for liver cancer therapy. N-terminal EF-hand calcium binding protein 3 (NECAB3) is a new recognized regulator of cancer, while its role in liver cancer remained elusive. Thus, the study clarified the action of NECAB3 on liver cancer development and explored the detailed mechanism. We found that NECAB3 was enhanced in liver cancer. Knockdown of NECAB3 restrained liver cancer cell migration and invasion. Besides, knockdown of NECAB3 suppressed the activation of the hypoxia-inducible factor 1-alpha (HIF-1α)/Ras like without CAAX 1 (RIT1) pathway. Furthermore, NECAB3 regulated liver cancer migration and invasion through modulating RIT1 expression. Moreover, downregulation of NECAB3 suppressed liver cancer tumor growth *in vivo*. In conclusion, NECAB3 was upregulated in liver cancer. Knockdown of NECAB3 suppressed aggressive phenotype of liver cancer via modulating the HIF-1α/RIT1 axis, providing a possible target for liver cancer therapy.

## 1 Introduction

Liver cancer is a prevalent malignant tumor globally [1,2]. In China, the morbidity of liver cancer is the fourth highest, and mortality is the second highest among cancers [3]. It is estimated that the number of people died from liver cancer will reach one million in 2030 [1,2,4]. Liver cancer is mainly caused by infection of hepatitis B virus or hepatitis C virus, alcohol-induced cirrhosis, and metabolic diseases [5]. Most liver cancer patients were diagnosed at last stage owing to the deficiency of effective diagnostic tools. Although advances have been made in therapeutic means including natural compounds, chemotherapy, immunotherapy, and combination therapy, the survival of advanced patients remains poor [6]. For example, the median overall survival of late-stage treatment drug *Lenvatinib* in advanced liver cancer patients was 13.6 months [7]. Therefore, liver cancer is a significant threat to global health. It is crucial to elucidate the mechanism of liver cancer development.

N-terminal EF-hand calcium binding protein 3 (NECAB3) is a cancer progression regulator discovered in recent years [8,9]. For instance, Nakaoka et al. found that NECAB3 promoted hypoxia-inducible factor-1 (HIF-1) activation during normoxia and drove tumorigenicity of cancer cells [8]. Li et al. reported that NECAB3 potentiated the aggressive phenotype of non-small cell lung cancer (NSCLC) cells via modulating macrophage polarization [9]. Besides, Zheng et al. found that NECAB3 was significantly upregulated in hepatocellular carcinoma based on the data of GSE14520 [10]. However, the action of NECAB3 on liver cancer remained elusive. Interestingly, the previous research proved that NECAB3 could activate hypoxia-inducible factor-1 alpha (HIF-1α) in cancer cells [8]. Meanwhile, HIF-1α was considered a critical transcription factor in cancer progression and target therapy for cancers [11]. For example, Liu et al. demonstrated that hypoxia-induced LncRNA-MIR210HG drove ovarian cancer progression by suppressing HIF-1α degradation [12]. Furthermore, in liver cancer, HIF-1α has been shown to modulate cancer growth and metastasis and cell sensitivity to Sorafenib through inducing Ras like without CAAX 1 (RIT1) expression [13]. Therefore, we inferred that the upregulated NECAB3 might regulate liver cancer progression via modulating the HIF-1α/RIT1 axis.

Hence, the study was aimed to elucidate the action of NECAB3 on liver cancer development and explore the detailed mechanism.

## 2 Materials and methods

### 2.1 Bioinformatical analysis

The differential expression of NECAB3 in liver cancer was analyzed using the clinical data of The Cancer Genome Atlas (TCGA) database. Data of 371 liver cancer tissues and 50 normal tissues were used for this analysis.

### 2.2 Cell culture

The human hepatocytes and liver cancer cells including Huh-7, Hep3B2, Li7, and HCCLM3 were obtained from Bena Culture Collection (Beijing, China). These cells were cultured in DMEM medium supplemented with 10% FBS at 37°C atmospheres with 5% CO_2_.

### 2.3 Quantitative real-time polymerase chain reaction (qRT-PCR)

RNA samples were obtained utilizing TRIzol (Beyotime, Shanghai, China). After quantification, the RNA samples (1 μg) were used for generating cDNA by High Capacity cDNA Reverse Transcription Kit, and qPCR assay was conducted via using PowerTrack™ SYBR Green Master Mix (Applied Biosystems, Waltham, MA). The primers of NECAB3 were: F: 5′-TCTGGCAGGATGAGGC-3′; R: 5′-GAGGCTGGG AAGAACAC-3′ [9]. *β-actin* was employed as the control. Relative NECAB3 expression was obtained by the 2^−ΔΔCt^ method.

### 2.4 Western blot

Cells and tissue lysates were obtained utilizing RIPA buffer (Beyotime, Shanghai, China), and quantified by the BCA method. The obtained protein samples (40 μg) were size-fractionated on SDS-PAGE gels and then transferred to PVDF membranes. After hindered non-specific binding, the membranes were probed with anti-NECAB3 (PA5-101422, 1:1,000, Thermo Fisher Scientific), HIF-1α (ab216842, 1:1,000, Abcam), RIT1 (ab53720, 1:1,000, Abcam), and actin (ab200658, 1:5,000, Abcam) antibodies, and then incubated with secondary antibody IgG H&L (HRP) (ab6721, Abcam, Cambridge, MA, UK). Eventually, the bands were observed through using the ECL detection system (Bio-Rad, Hercules, CA, USA).

### 2.5 Cell transfection

The siRNA against NECAB3 (siNECAB3), overexpression plasmid of NECAB3, siRNA against RIT1 (siRIT1), overexpression vector of RIT1, and negative controls were constructed by Sangon Biotech (Shanghai, China). Lipofectamine 3000 (Invitrogen, Waltham, MA, USA) was applied to transfect the above plasmids in the manner of the supplier’s protocols. After 48 h, the cells were harvested to perform the subsequent experiments.

### 2.6 Wound healing assay

Cells were seeded into six-well plates (5 × 10^5^ cells/well) and cultured to full confluency. The wound was made in cell monolayer using a 200 μL pipette tip, and cells were grown for 24 h. Then, the wound healing results were obtained using a microscope.

### 2.7 Transwell invasion assay

The upper chamber of transwell chambers pre-coated with Matrigel was plated into 1 × 10^5^ cells with a serum-free medium. The complete medium was added to the lower chamber. Then, the chambers were placed at 37°C with 5% CO_2_ for 24 h. After non-invaded cells were removed, the cells were immobilized, dyed with crystal violet, and analyzed using the microscope.

### 2.8 Xenograft tumor establishment

The animal experiments were conducted following guidelines of the National Institutes of Health and authorized by the Ethics Committee of Suzhou Science & Technology Town Hospital. Twelve BALB/c nude mice aged 6 weeks were selected to establish xenograft tumor. 1 × 10^7^ Li7 cells, stably transfected with shNECAB3 or shNC vector were subcutaneously injected into mice. The tumor volumes were monitored after 7 days post injection until 35 days. Tumor volume was determined utilizing the formula: *V* = (Length × Width^2^)/2. Then, mice were anesthetized, and tumors were separated to determine the expression of NECAB3, HIF-1α, and RIT1.

### 2.9 Immunohistochemistry

Tumor tissues were fixed, embedded, and sectioned (5 μm). After being deparaffinized, rehydrated, and blocked, the samples were probed to anti-NECAB3 antibody (PA5-101422, 1:200, Thermo Fisher Scientific) at 4°C overnight, and then treated with IgG H&L (HRP) (ab6721, Abcam, Cambridge, MA, UK) and stained with the DAB (R&D Systems, Minneapolis, MN, USA). The dyed pictures were taken using the microscope.

### 2.10 Statistical analysis

Data were expressed as mean value ± standard deviation (SD). SPSS Statistics 22.0 (SPSS, Chicago, IL, USA) was used to accomplish the analysis of group differences with Student’s *t*-test and one-way ANOVA methods. *P* < 0.05 was noted as statistically significant.


**Ethics approval:** Ethical approval was obtained from the Ethics Committee of Suzhou Science & Technology Town Hospital.

## 3 Results

### 3.1 NECAB3 was highly expressed in liver cancer

**Figure 1:**
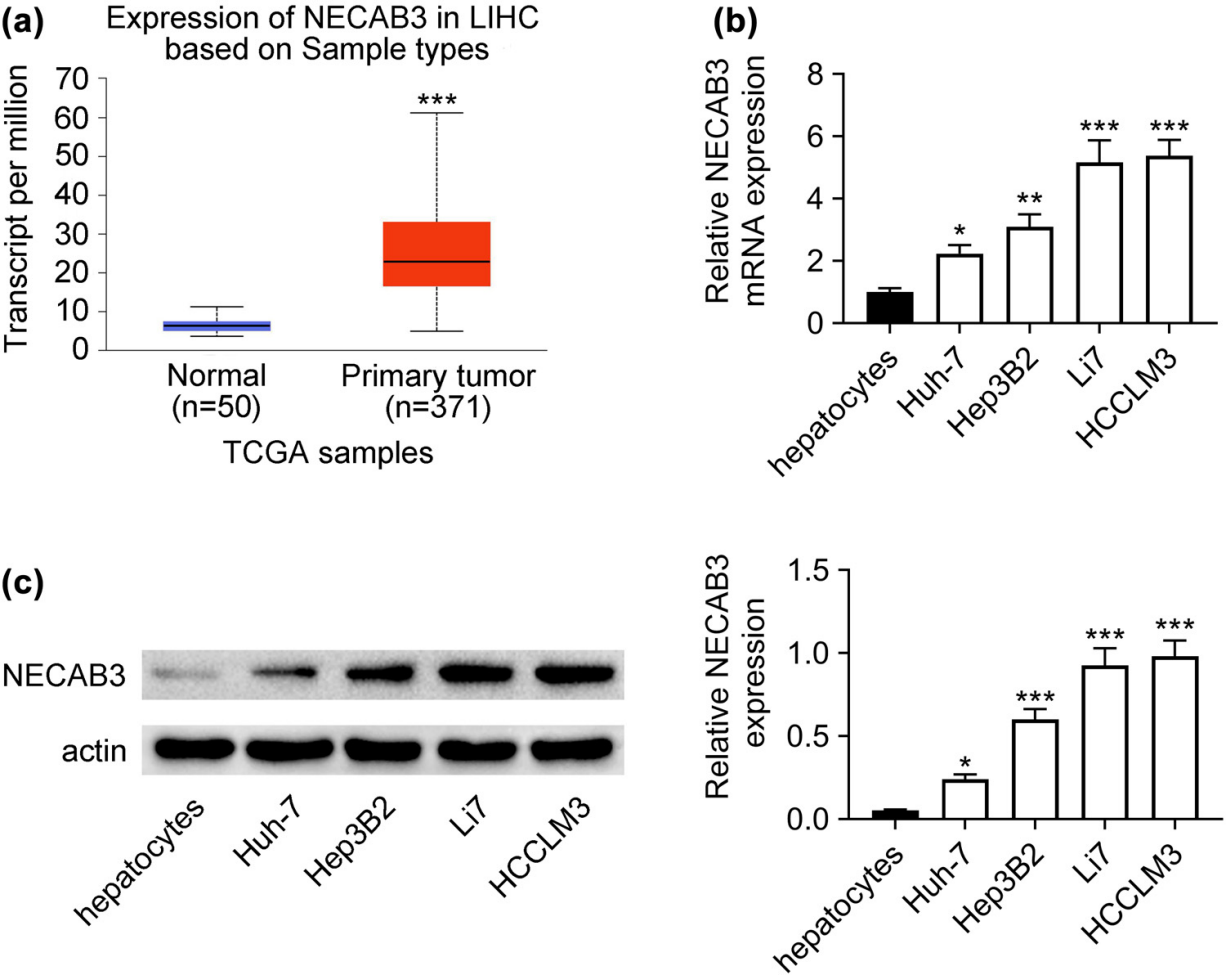
The expression of NECAB3 was upregulated in liver cancer. (a) The expression of NECAB3 was analyzed using the data of 371 liver cancer tissues and 50 normal tissues from TCGA. (b) NECAB3 mRNA expression in human hepatocytes and liver cancer cell lines including Huh-7, Hep3B2, Li7, and HCCLM3 were determined using qRT-PCR. (c) NECAB3 protein level in human hepatocytes and liver cancer cell lines including Huh-7, Hep3B2, Li7, and HCCLM3 were detected by Western blot. *: *P* < 0.05. **: *P* < 0.01. ***: *P* < 0.001.

To elucidate the differential expression of NECAB3 in liver cancer, the data of 371 liver cancer tissues and 50 normal tissues from TCGA were analyzed. It was observed that NECAB3 was significantly elevated in liver cancer tissues (*P* < 0.001, Figure 1a). Besides, the NECAB3 expression in liver cancer cells including Huh-7, Hep3B2, Li7, and HCCLM3 and human hepatocytes was detected. Results revealed that NECAB3 was highly expressed in liver cancer cells (*P* < 0.05, Figure 1b and c). Therefore, NECAB3 was upregulated in liver cancer.

### 3.2 Knockdown of NECAB3 suppressed the migrative and invasive ability of liver cancer cells

**Figure 2:**
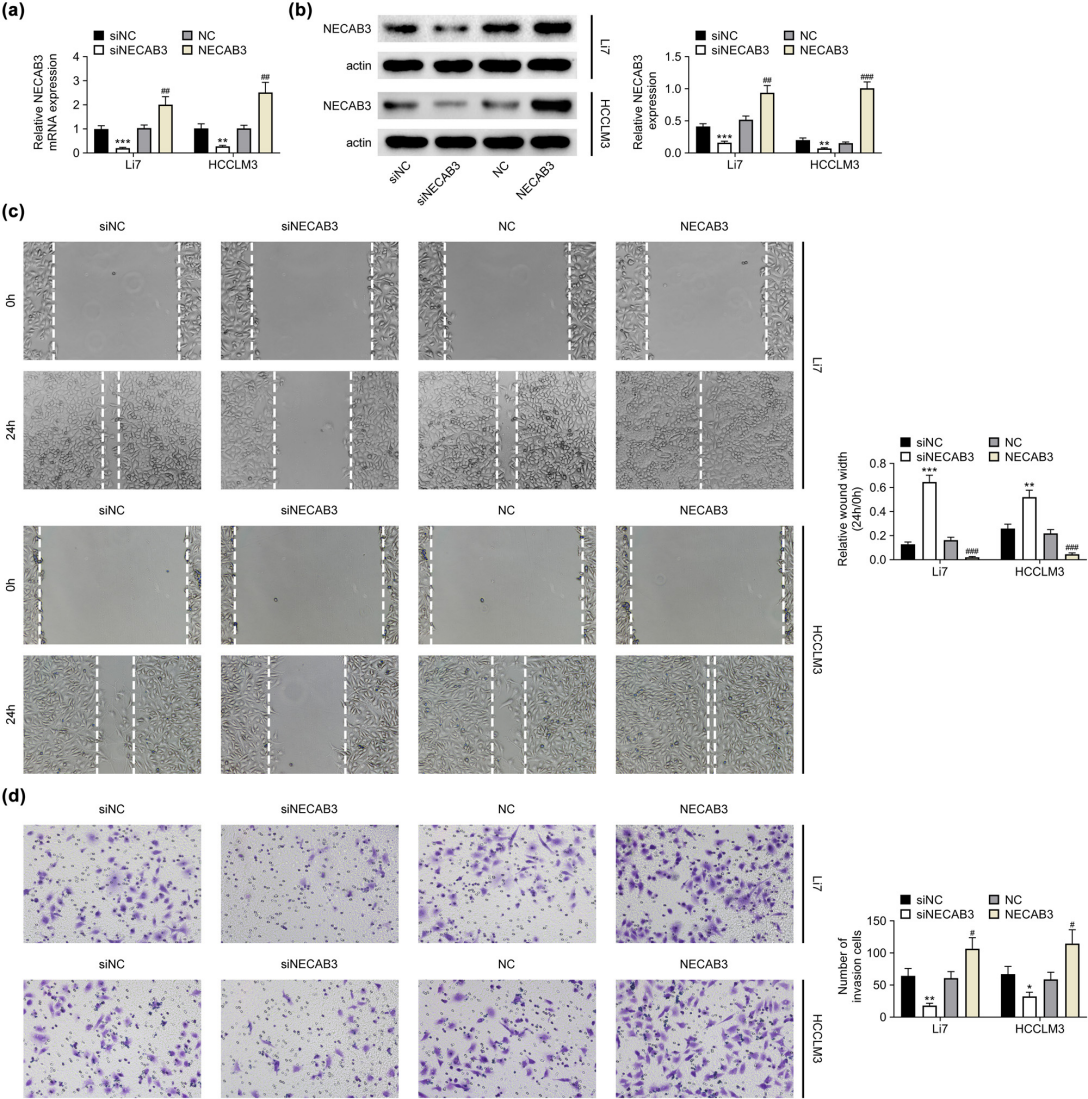
Knockdown of NECAB3 suppressed the migrative and invasive ability of liver cancer cells. (a) NECAB3 mRNA expression in Li7 and HCCLM3 cells transfected with siNECAB3 and overexpression vector of NECAB3 was measured by qRT-PCR. (b) The protein level of NECAB3 in Li7 and HCCLM3 cells transfected with siNECAB3 and overexpression vector of NECAB3 was measured by Western blot. (c) Wound healing assay was used to assess the migration ability of Li7 and HCCLM3 cells transfected with siNECAB3 and overexpression vector of NECAB3. (d) Transwell invasion assay evaluated the invasion ability of Li7 and HCCLM3 cells transfected with siNECAB3 and overexpression vector of NECAB3. *: *P* < 0.05. **: *P* < 0.01. ***: *P* < 0.001. #: *P* < 0.05. ##: *P* < 0.01. ###: *P* < 0.001.

To clarify the biological function of NECAB3 in liver cancer, NECAB3 expression was altered through transfection of siNECAB3 and overexpression plasmid of NECAB3 in Li7 and HCCLM3 cells. Results found that siNECAB3 significantly knocked down the NECAB3 expression, while overexpression plasmid of NECAB3 obviously increased NECAB3 expression in Li7 and HCCLM3 cells (*P* < 0.01, Figure 2a and b). Besides, downregulation of NECAB3 suppressed the migrative ability of liver cancer cell, whereas upregulated NECAB3 exerted the opposite function (*P* < 0.01, Figure 2c). The invasive ability of liver cancer cells was impaired by downregulation of NECAB3 and enhanced by increased NECAB3 (*P* < 0.05, Figure 2d). Thus, the knockdown of NECAB3 restrained the migrative and invasive ability of liver cancer cells.

### 3.3 Knockdown of NECAB3 suppressed the activation of the HIF-1α/RIT1 pathway

**Figure 3:**
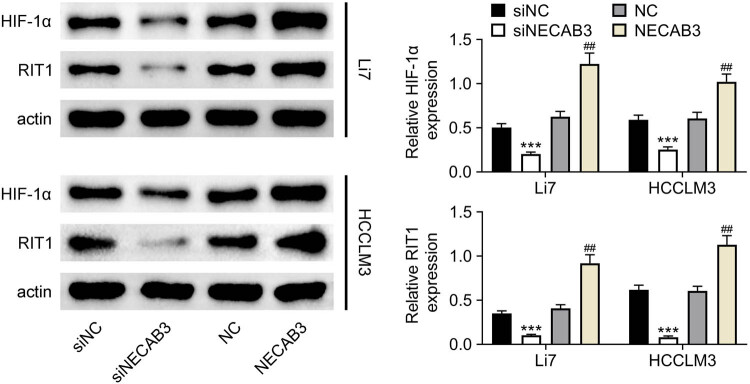
Knockdown of NECAB3 suppressed the activation of the HIF-1α/RIT1 pathway. The protein levels of HIF-1α and RIT1 in Li7 and HCCLM3 cells transfected with siNECAB3 and overexpression vector of NECAB3 were analyzed by Western blot. ***: *P* < 0.001. ##: *P* < 0.01.

To investigate the mechanism of NECAB3 on liver cancer progression, the activation of the HIF-1α/RIT1 pathway was determined after the expression of NECAB3 was altered. The result showed that downregulation of NECAB3 inhibited the expression of HIF-1α and RIT1 in Li7 and HCCLM3 cells (*P* < 0.01, Figure 3). Contrarily, enforced NECAB3 considerably increased the HIF-1α and RIT1 levels (*P* < 0.01, Figure 3). Hence, silencing NECAB3 suppressed the activation of the HIF-1α/RIT1 pathway.

### 3.4 NECAB3 regulated the liver cancer progression through modulating RIT1

**Figure 4:**
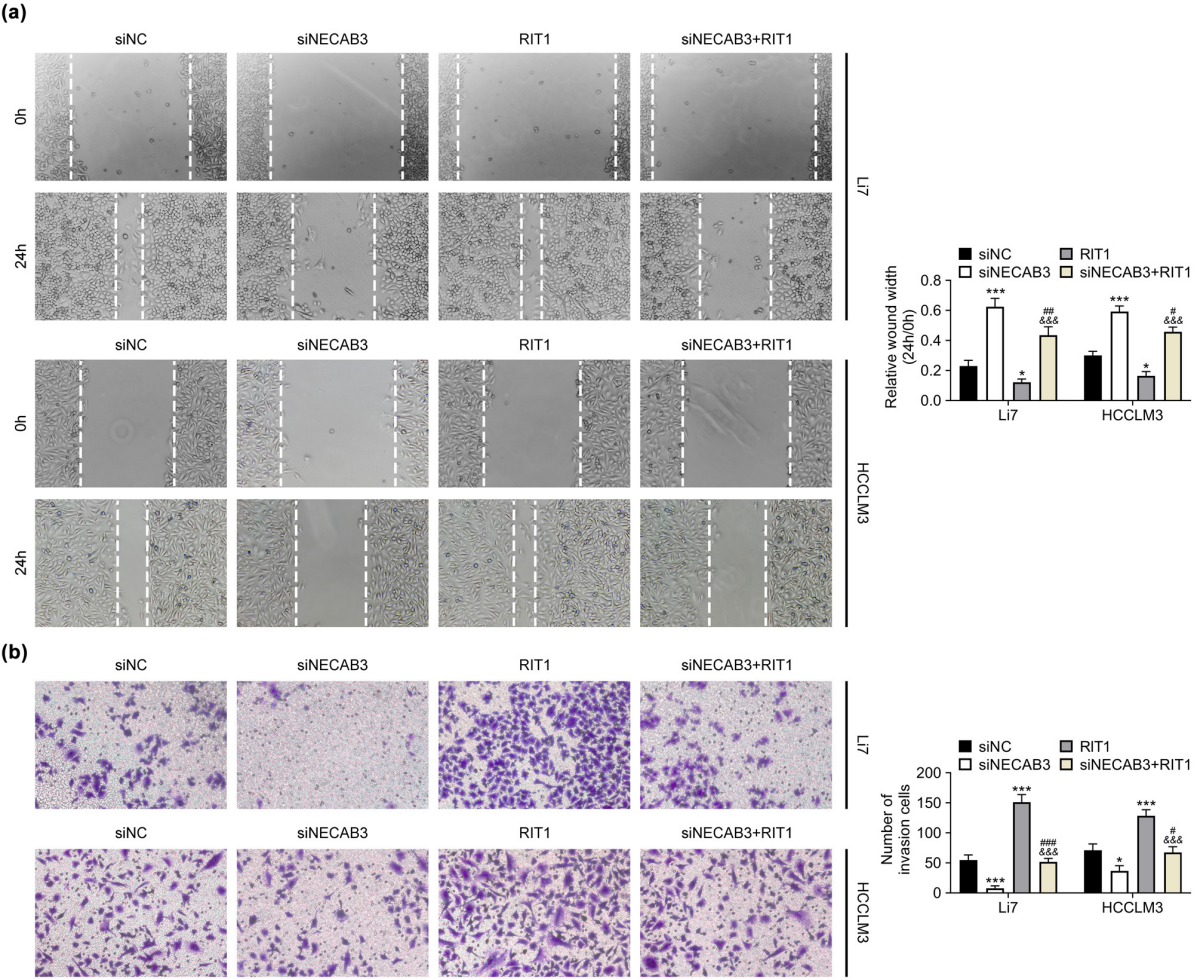
Knockdown of NECAB3 suppressed liver cancer progression through downregulating RIT1. (a) Wound healing assay was used to assess the migrative ability of Li7 and HCCLM3 cells co-transfected with siNECAB3 and overexpression vector of RIT1. (b) Transwell invasion assay was used to evaluate the invasive ability of Li7 and HCCLM3 cells co-transfected with siNECAB3 and overexpression vector of RIT1. *: *P* < 0.05. ***: *P* < 0.001. #: *P* < 0.05. ##: *P* < 0.01. ###: *P* < 0.001. &&&: *P* < 0.001.

**Figure 5:**
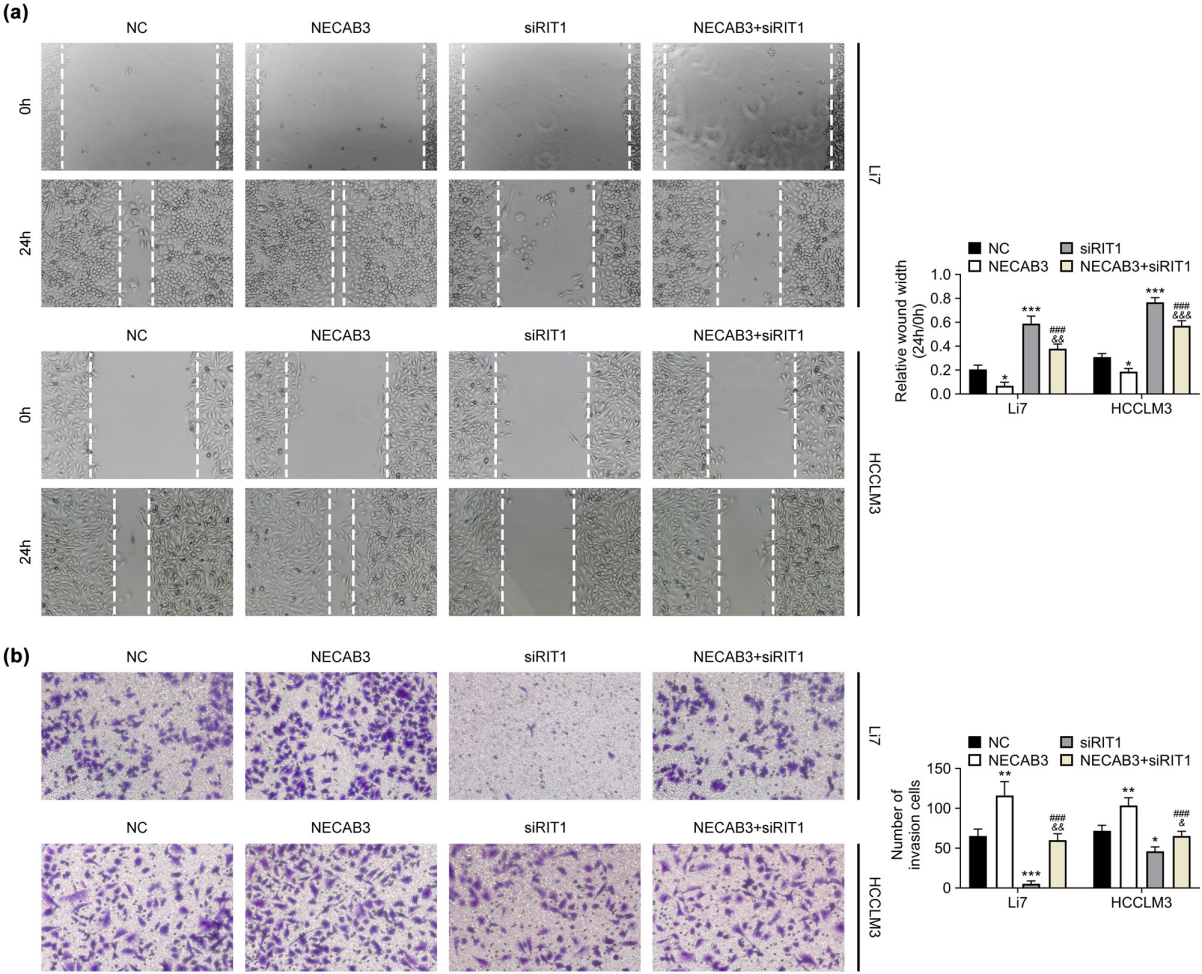
Overexpression of NECAB3 accelerated liver cancer progression through upregulating RIT1. (a) Wound healing assay was used to assess the migrative ability of Li7 and HCCLM3 cells co-transfected with overexpression vector of NECAB3 and siRIT1. (b) Transwell invasion assay was used to evaluate the invasive ability of Li7 and HCCLM3 cells co-transfected with overexpression vector of NECAB3 and siRIT11. *: *P* < 0.05. **: *P* < 0.01. ***: *P* < 0.001. ###: *P* < 0.001. &: *P* < 0.05. &&: *P* < 0.01. &&&: *P* < 0.001.

To study the role of NECAB3 in regulating liver cancer, siNECAB3 and overexpression plasmid of RIT1 were co-transfected into Li7 and HCCLM3 cells. Then, functional experiments revealed that downregulation of NECAB3 suppressed liver cancer cell migration and overexpression of RIT1 promoted cell migration (*P* < 0.05, Figure 4a). Besides, cell migration inhibited by downregulation of NECAB3 was reversed by enforced RIT1 (*P* < 0.05, Figure 4a). Additionally, knockdown of NECAB restrained liver cancer cell invasion and overexpression of RIT1 promoted cell invasion (*P* < 0.05, Figure 4b). The invasive ability impaired by knockdown of NECAB3 was reversed by overexpression of RIT1 (*P* < 0.05, Figure 4b). Furthermore, overexpression plasmid of NECAB3 and siRIT1 were also co-transfected into Li7 and HCCLM3 cells. As expected, it was observed that overexpression of NECAB3 enhanced liver cancer cell migration and invasion, which was suppressed by downregulation of RIT1 (*P* < 0.05, Figure 5a and b). These findings suggested that NECAB3 regulated liver cancer progression through modulating RIT1.

### 3.5 Downregulation of NECAB3 suppressed liver cancer tumor growth *in vivo*

**Figure 6:**
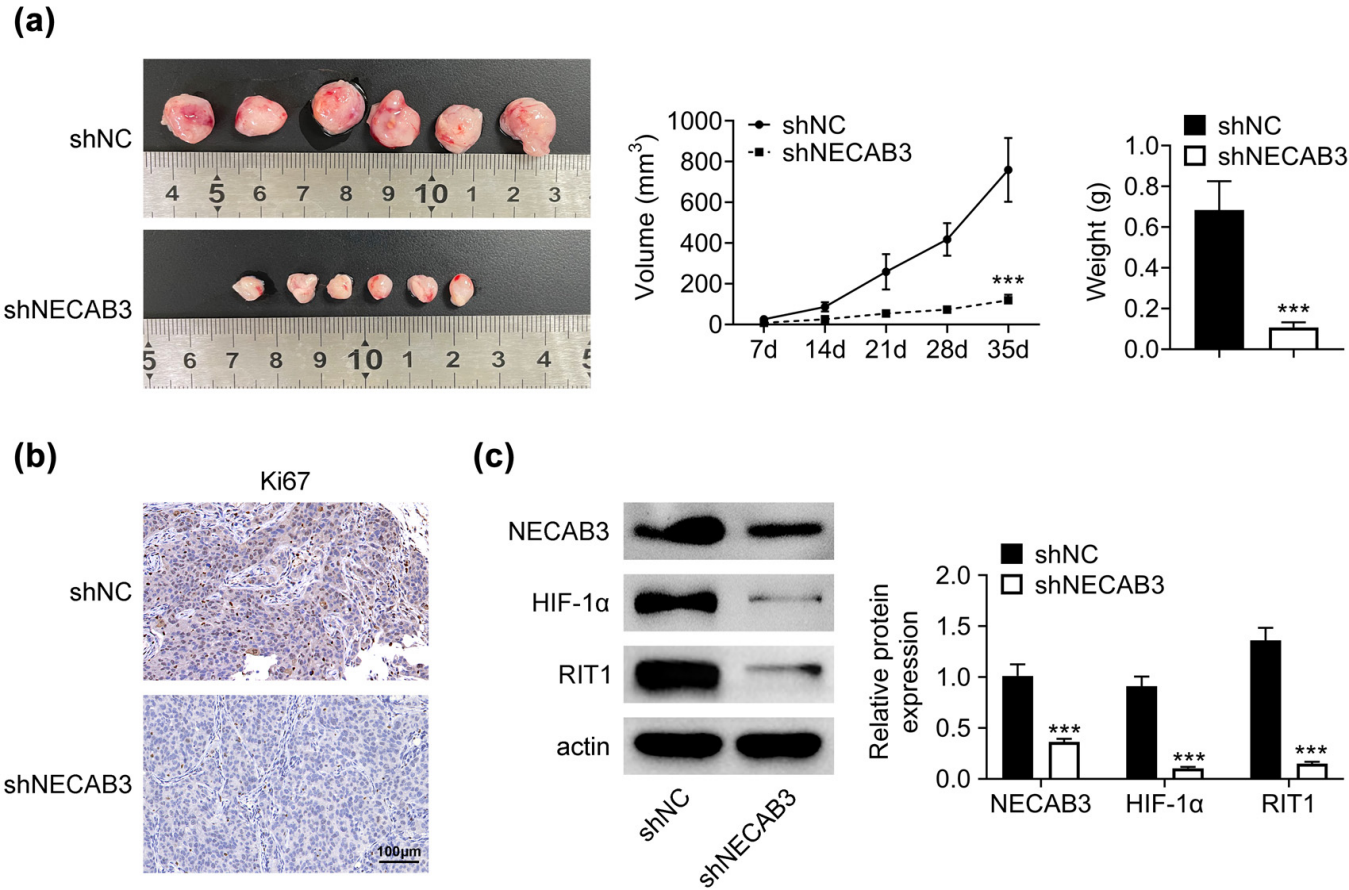
Down-regulation of NECAB3 suppressed liver cancer tumor growth *in vivo*. (a) The images of xenograft tumor established using Li7 cells, which were stably transfected with shNECAB3, and the tumor volume was monitored. (b) Immunohistochemistry was used to detect the expression of NECAB3 in tumor tissues. (c) Western blot was used to measure the protein levels of NECAB3, HIF-1α, and RIT1 in tumor tissues. ***: *P* < 0.001.

To better elucidate the action of NECAB3 on liver cancer *in vivo*, xenograft tumor was constructed utilizing Li7 cells, which were stably transfected with shNECAB3. Results revealed that downregulation of NECAB3 significantly suppressed tumor growth *in vivo* compared to the shNC group (*P* < 0.001, Figure 6a). Besides, immunohistochemistry results revealed that NECAB3 was inhibited in tumor tissues by shNECAB3 (Figure 6b). Furthermore, Western blot result showed that shNECAB3 greatly suppressed the protein levels of NECAB3, HIF-1α, and RIT1 in tumor tissues (*P* < 0.001, Figure 6c). In summary, downregulation of NECAB3 suppressed liver cancer tumor growth *in vivo*.

## 4 Discussion

NECAB3 is a newly recognized regulator of cancer progression [8,9], while its role in liver cancer remains to be elucidated. Hence, we emphasized the role of NECAB3 in liver cancer tumorigenesis in this study. To clarify the role of NECAB3 in liver cancer, the NECAB3 expression in liver cancer was detected. It was found that NECAB3 was upregulated in liver cancer. The finding was in agreement with the previous studies [9]. Li et al. showed that the expression of NECAB3 was dramatically enhanced in NSCLC cells [9]. Zheng et al. found that NECAB3 was significantly upregulated in hepatocellular carcinoma based on the data of GSE14520 [10]. Besides, the level of NECAB3 in Li7 and HCCLM3 cells was higher than that in Huh-7 cells in this study. The Huh7 cell line was well differentiated and presented low metastatic potential [14,15], while Li7 and HCCLM3 cells were highly metastatic cell lines [16,17]. Therefore, this finding suggested that NECAB3 expression was increased with the malignant degree of liver cancer cell lines.

The previous study revealed that NECAB3 played a vital role in the metabolism of β-amyloid [18]. Recent studies reported that NECAB3 also exerted regulation effect on cancer [8,9]. Given the aberrant expression of NECAB3 in liver cancer, we speculated that NECAB3 might modulate the liver cancer progression. As expected, this study revealed that silence of NECAB3 suppressed the migration and invasion of liver cancer cells, and overexpression of NECAB3 promoted the cancer cell migration and invasion. Similarly, NECAB3 was proved to promote tumor progression of NSCLC [9]. Nakaoka et al. also showed that NECAB3 depletion could suppress glycolysis and tumorigenicity [8].

HIF-1α was a critical transcription factor in cancer progression. Lin et al. discovered that increasing the stability of HIF-1α could enhance cell invasive ability in head and neck squamous cell carcinoma [19]. Zhang et al. found that HIF-1α was essential for activation and tumor-promotional effect of cancer-associated fibroblasts in lung cancer [20]. In this research, we found that silence of NECAB3 suppressed the HIF-1α expression in liver cancer cells and overexpression of NECAB3 increased HIF-1α expression. Similarly, the previous study showed that the activation of HIF-1α could be affected by NECAB3 [8]. NECAB3 mutants that binds Mint3 but lacks an intact monooxygenase domain also inhibited HIF-1 activation [8]. Furthermore, Song et al. reported that HIF-1α regulated liver cancer growth and metastasis through inducing RIT1 expression [13]. Therefore, the expression of RIT1 was determined in this study. It was found that decreased NECAB3 suppressed the expression of RIT1. RIT1 is a member of the Ras superfamily of small GTPases, and it functioned as oncogenic protein in cancer development [21]. In endometrial cancer, elevated expression of RIT1 was associated with poor prognosis [22]. In esophageal squamous cell carcinoma, RIT1 suppressed tumor cell growth and metastasis [23]. The RIT1 gene was amplified, which may be one of the activation ways in hepatocellular carcinoma [24]. Given the evidence, we speculated that NECAB3 might regulate liver cancer progression via modulating the HIF-1α/RIT1 axis. Consistent with the conjecture, results revealed that the inhibitory effect of downregulation of NECAB3 on liver cancer migration and invasion was reversed by the overexpression of RIT1. In other words, NECAB3 regulated liver cancer migration and invasion through modulating RIT1. Moreover, the *in vivo* experiments revealed that downregulation of NECAB3 suppressed liver cancer tumor growth, which indicated that NECAB3 may be a potential therapeutic target for treating liver cancer.

In conclusion, we reported the function and mechanism of NECAB3 regulating liver cancer progression for the first time. NECAB3 was upregulated in liver cancer. Knockdown of NECAB3 suppressed the aggressive phenotype of liver cancer via modulating the HIF-1α/RIT1 axis, providing a possible target for liver cancer therapy.
